# The diagnostic effectiveness of serum sialic acid predicts both qualitative and quantitative prostate cancer in patients with prostate-specific antigen between 4 and 20 ng/mL

**DOI:** 10.3389/fendo.2023.1188944

**Published:** 2023-08-14

**Authors:** Jingtao Sun, Lei Yan

**Affiliations:** Department of Urology, Qilu Hospital of Shandong University, Jinan, China

**Keywords:** serum sialic acid, prostate cancer, prostate biopsy, diagnosis, treatment

## Abstract

**Introduction:**

This study aimed to evaluate the predictive value of the serum biochemical index, including alkaline phosphatase (AKP), lactate dehydrogenase (LDH), α-L-fucosidase (AFU), serum sialic acid (SA), and fibrinogen (FIB), for prostate cancer (PCa) and clinically significant prostate cancer (CSPCa) in patients with a prostate-specific antigen (PSA) value between 4 and 20 ng/mL.

**Patients and methods:**

This study retrospectively examined the clinical data of 408 eligible patients who underwent prostate biopsies in our hospital between March 2015 and July 2022. CSPCa was defined as a “Gleason grade group of≥2”. For analyzing the association between PCa/CSPCa and serum biochemical index, univariable logistic regression and multivariable logistic regression were conducted. Based on the multivariable logistic regression model, we constructed models and compared the area under the curve (AUC). We generated the nomogram, the ROC curve, the DCA curve, and the calibration curve for PCa.

**Results:**

Overall, we studied 271 patients with PCa (including 155 patients with CSPCa) and 137 non-PCa patients. Patients with PCa were more likely to consume alcohol, have higher total PSA (TPSA) values, and have lower free PSA (FPSA) and free/total PSA (f/T) values. There were higher TPSA values and lower f/T values in the CSPCa group when compared with the non-CSPCa group. The univariate logistic regression analyses did not show significant results. However, AKP, AFU, SA, TPSA, and FPSA all retain significant significance when all factors are included in multifactor logistic regression analysis. This finding suggests that the exposure factor exhibited an independent effect on the outcome after controlling for other factors, including the potential confounding effects that may have been underestimated. Through ROC curves, we found that SA and TPSA levels are more powerful predictors. In contrast, there is a lack of excellent predictive value for PCA and CSPCa using Age, AFU, FIB, and FPSA.

**Conclusion:**

In our study, serum biochemical index is a potential prediction tool for PCa and CSPCa for patients with PSA values between 4 and 20 ng/mL. Additionally, the new serum biochemical index SA is also useful when diagnosing PCa and CSPCa, as we conclude in our study.

## 1 Introduction

Worldwide, prostate cancer is the second leading cause of cancer-related death among men after lung cancer (1). The incidence of prostate cancer continued to rise slowly from 2014 through 2018. Over the past decade, the proportion of prostate cancer cases diagnosed at a distant stage has increased from 3.9% to 8.2% (2). Prostate-specific antigen (PSA) is an important biomarker for detecting prostate cancer. But PSA tests are not sensitive enough to detect prostate cancer early (PCA) because of their low specificity (3). In other words, most patients undergo unnecessary and potentially harmful follow-up tests, like biopsies, especially if their PSA level is between 4.0 and 20.0 ng/ml (4). The need for more specific biomarkers is, therefore, necessary to compensate for this defect. Many biomarkers of PCa have been developed successively, such as Prostate Health Index (PHI), prostate cancer antigen 3 (PCA3), four-kallikrein panel (4K), transmembrane protease serine 2-ERG (TMPRSS2-ERG), ExoDx Prostate Intelliscore and SelectMDx (5, 6). There are, however, a few disadvantages to these experimental methods, making them unsuitable for routine PCa detection (7).

Certain systemic serum biochemical indexes have been recognized as being important in promoting and advancing tumor progression in recent years. Tumor-related serum biochemical indexes, including alkaline phosphatase (AKP), lactate dehydrogenase (LDH), α-L-fucosidase (AFU), serum sialic acid (SA), and fibrinogen (FIB), have gained attention as diagnostic tools for tumors (8–10). Despite this, the study on the effects of multiple biochemical indices combined on tumor growth is not comprehensive.

In glycoproteins and glycolipids, SA is a series of hydroxylated monosaccharides containing nine carbon atoms at methylated non-reduction terminals In glycoproteins and glycolipids, SA is a series of hydroxylated monosaccharides containing nine carbon atoms at the methylated non-reduction terminals (11). There has been substantial evidence to demonstrate that cancer is associated with high serum levels of sialic acid, which have been seen in cancer patients in numerous studies (12), for instance, oral cancer (13), breast cancer (14), ovarian cancer (15) and cholangiocarcinoma (16).

A primary objective of our study was to investigate whether the SA could be used at the PSA level of 4.0 to 20.0 ng/mL to predict PCa and CSPCa. We validated the diagnostic efficacy of the AKP, LDH, AFU, and FIB in PCa and CSPCa as well.

## 2 Materials and methods

### 2.1 Patient selection information collection

From March 2015 to July 2022, we obtained information from the electronic medical record system about all patients who underwent prostate biopsies with PSA levels of 4.0-20.0ng/mL in our hospital. Blood tests were performed within 2 weeks before biopsies on all patients for the serum PSA derivative (TPSA and fPSA). Our study excluded patients with any one or more of the following conditions: (I) Patients with hematological diseases, known infections, and other malignancies; (II) Patients who had undergone prostate surgery (such as transurethral resection) before their biopsies; (III) Pathologically diagnosed patients with prostatic intraepithelial neoplasms and atypical small acinar proliferations; (IV) Patients with incomplete clinical data. Following that, we collected the following information from the medical records of eligible patients: age, history of tobacco (SH) and alcohol use (AH), blood test results with alkaline phosphatase (AKP), lactate dehydrogenase (LDH), α-L-fucosidase (AFU), fibrinogen (FIB), and prostate-specific antigen (PSA).

### 2.2 Blood biochemical measurement

Before receiving any clinical intervention, each patient had his venous blood drawn in the early morning after fasting for 12 hours. We stored blood samples in test tubes that contained clot activator and gel. Blood samples coagulated naturally at room temperature during the experiment. Following that, the samples were centrifuged at 2000 rpm for 10 minutes. A Roche Cobas 8000 automatic analyzer was used to determine SA concentrations after the serum had been separated. AKP concentrations in the normal range ranged from 45.00 U/dL to 125.00 U/L.The normal range for LDH concentration was 120.00 U/dL to 230.00 U/L. In terms of AFU concentration, the normal range was less than 40.00 U/L. The concentration of SA was normally between 45.6 mg/dL and 75.4 mg/dL.

### 2.3 Fibrinogen measurement

Surgical procedures are routinely preceded by the collection of blood samples from patients to measure plasma fibrinogen levels. Fibrinogen levels in the blood were determined by the Clauss (17) method using bovine thrombin (100 NIHU/mL). Plasma fibrinogen levels between 2-4 g/L were considered normal.

### 2.4 Biopsy method and pathological examination

Using 3.0 T scanner, two uroradiologists with at least three years of experience performed prostate mpMRI before undergoing biopsies. Imaging assessments were retrospectively performed by experienced surgical team members to determine biopsy methods based on imaging findings. Lastly, local anesthesia was used to perform transrectal biopsies or transperineal biopsies on all patients. Prostate biopsies incorporated 12 + 3 cores (based on 12 systematic cores, and the remaining core at the suspicious MRI area that was visualized by cognitive fusion biopsies). After the biopsy specimens were collected, they were analyzed within a week by two experienced urologists following the ISUHP (International Society of Urological Pathology) consensus guidelines.

### 2.5 Data management

Based on the histopathological results, patients were categorized as non-PCa groups or PCa groups. Moreover, we separated the patients into CSPCa and non-CSPCa groups. It was defined as “clinically significant prostate cancer (CSPCa)” when referring to the Gleason grade group of≥2. After fasting for 12 hours, each patient was given five milliliters of venous blood in the early morning before any clinical intervention was carried out. To test the clot activator and gel, a blood sample was stored at room temperature and naturally coagulated. After centrifugation at 2000 rpm for 10 minutes, the samples were collected.

### 2.6 Statistical analysis

Normality tests were conducted on all continuous variables. In the case of continuous variables that passed the normality test, Student’s t-tests were used, whereas Mann-Whitney U-tests were used in the case of continuous variables with skewed distributions. For continuous variables with normal distribution, the mean+SD was reported, and for continuous variables with skewed distribution, the median (IQR) was reported. Numbers (percentages) were reported for categorical variables after Chi-square tests were conducted. We performed univariate and multivariate logistic regression analyses to identify the independent predictors of PCa and CSPCa. The final model selection was performed using a backward stepdown selection process. Significant results were determined by a p-value of less than 0.05. An analysis of the ROC curve, DCA curve, and calibration curves was carried out to determine the validity of the PCa risk nomogram we developed for prostate biopsy. Differences in AUC were compared with the DeLong test. Statistical analyses were conducted using IBM SPSS (Version 19.0) and R (Version 4.1.0) software.

## 3 Results

### 3.1 Patient demographics eligible for participation in the program

The study included a total of 408 patients who met the entry criteria. 271 patients were diagnosed with PCa (including 155 patients with CSPCa). As shown in **Table 1**, the patients were classified according to their characteristics and laboratory values.

**Table 1:** Characteristic baseline.

Variable (n=408)	Non-PCa (n=137)	PCa (n=271)	p Value	Non-CSPCa (n=253)	CSPCa (n=155)	p Value
Age, year	68.00(63.00,73.00)	68.00(63.00,74.00)	0.99	69.00(63.00,74.00)	68.00(62.00,74.00)	0.33
SH (%)			0.04			0.84
Y	54.00(39.40)	80.00(29.50)		84.00(33.20)	50.00(32.30)	
N	83.00(60.60)	191.00(70.50)		169.00(66.80)	105.00(67.70)	
AH (%)			0.10			0.46
Y	51.00(37.20)	79.00(29.20)		84.00(33.20)	46.00(29.70)	
N	86.00(62.80)	192.00(70.80)		169.00(66.80)	109.00(70.30)	
AKP,U/L	70.00(60.00,82.00)	66.50(56.25,78.00)	0.10	68.00(56.00,79.00)	67.00(58.25,82.00)	0.47
LDH,U/L	192.00(173.00,221.00)	187.50(168.00,211.00)	0.22	186.00(168.75,211.00)	194.00(172.00,218.00)	0.08
AFU,U/L	17.00(13.00,22.00)	17.00(13.25,21.00)	0.94	17.00(13.00,21.50)	17.00(14.00,22.00)	0.61
SA,mg/dL	57.70(51.40,66.60)	53.60(49.73,59.73)	<0.01	54.70(50.60,62.20)	54.30(48.88,60.65)	0.15
FIB,g/L	3.17(2.75,3.63)	3.00(2.65,3.42)	0.07	2.98(2.62,3.48)	3.11(2.70,3.49)	0.26
TPSA,ng/mL	8.27(6.24,11.42)	10.49(7.72,13.76)	<0.01	8.74(6.91,11.89)	10.88(8.01,14.86)	<0.01
FPSA,ng/mL	1.41(1.03,1.80)	1.24(0.80,1.83)	0.02	1.29(0.93,1.76)	1.28(0.80,2.00)	0.66
f/T	0.18(0.13,0.25)	0.12(0.08,0.18)	<0.01	0.16(0.11,0.21)	0.12(0.08,0.17)	<0.01

Data are presented as median (P25, P75) or n (%). SH, smoking history; AH, alcohol history; AKP, Alkaline phosphatase; LDH, Lactate dehydrogenase; AFU, α-L-fucosidase; SA, Serum sialic acid; FIB, Fibrinogen; TPSA, total prostatic specifific antigen; fPSA, free prostatic specifific antigen; f/T, free/total prostatic specifific antigen ratio; PCa, prostate cancer; CSPCa, clinically signifificant prostate cancer, which was defifined as Gleason grade ≥ 2.

Besides TPSA (8.27 vs 10.49, p<0.01), the SA (57.70 vs 53.60, p<0.01), FPSA (1.41 vs 1.24, p=0.02) and f/T (0.18 vs 0.25, p<0.01) of the non-PCa group were significantly higher than those of the PCa group. Furthermore, PCa patients also had a higher proportion of smokers in their group as compared to non-PCa patients (**Table 1**).

As compared to the non-CSPCa group, the CSPCa group had higher levels of LDH and TPSA. In contrast, CSPCa groups had lower f/T values. In terms of SH, SA, and FPSA, however, there was no significant difference between the two groups. Further, there were no statistically significant differences in age, AH, AFU, and FIB between PCa and non-PCa groups, nor between CSPCa and non-CSPCa groups (**Table 1**).

### 3.2 Univariable and multivariable analyses of clinical indicators

In order to determine the predictive factors for clinical indicators, we conducted both univariate and multivariate logistic regression analyses. Furthermore, we conducted a collinearity analysis in multiple regression to comprehensively assess the variables that could be included in the model. The results of the collinearity analysis indicate that there is no interaction among the factors. The univariate logistic regression analyses yielded insignificant findings, with the exception of smoking history and TPSA level. The final model selection was made using a backward stepdown selection process. Significantly, AKP, AFU, SA, TPSA, and FPSA maintained statistical significance when subjected to multifactor logistic regression analysis that included all factors (refer to **Table 2**, PCa vs non-PCa, all VIF < 5.000). This finding suggests that the exposure factor exhibited an independent effect on the outcome after controlling for other factors, including the potential confounding effects that may have been underestimated.

**Table 2:** Univariable and multivariable analyses of clinical indicators.

PCa	Colinearity	Univariable Regression Analysis		Multivariable Regression Analysis		CSPCa	Univariable Regression Analysis		Multivariable Regression Analysis	
	VIF	OR (95% CI)	*p* Value	OR (95% CI)	*p* Value		OR (95% CI)	*p* Value	OR (95% CI)	*p* Value
Age, year	1.050	0.999(0.973-1.025)	0.92	/	/		0.984(0.959-1.010)	0.218	/	/
SH (%)	1.759	0.644(0.418-0.990)	0.045	/	/		0.958(0.625-1.468)	0.844	/	/
Y										
N										
AH (%)	1.745	0.694(0.449-1.071)	0.099	/	/		0.849(0.551-1.309)	0.459	/	/
Y										
N										
AKP,U/L	1.095	1.002(0.997-1.007)	0.484	/	/		1.001(0.997-1.005)	0.633	/	/
LDH,U/L	1.056	0.998(0.993-1.003)	0.398	/	/		1.004(0.999-1.009)	0.119	/	/
AFU,U/L	1.055	1.009(0.973-1.045)	0.637	1.086(1.028-1.147)	0.003		1.016(0.980-1.052)	0.390	1.063(1.018-1.110)	0.005
SA,mg/dL	1.539	0.997(0.989-1.005)	0.493	0.896(0.855-0.938)	0.000		1.003(0.995-1.011)	0.517	0.944(0.910-0.980)	0.003
FIB,g/L	1.505	0.947(0.768-1.168)	0.613	2.187(1.169-4.092)	0.014		1.087(0.885-1.334)	0.426	2.160(1.243-3.754)	0.006
TPSA,ng/mL	1.245	1.183(1.082-1.293)	0.000	1.184(1.083-1.293)	0.000		1.115(1.060-1.173)	0.000	1.134(1.064-1.209)	0.000
FPSA,ng/mL	1.353	0.860(0.709-1.042)	0.124	0.739(0.556-0.980)	0.036		1.062(0.879-1.282)	0.534	/	/
F-PSA/PSA	1.027	0.937(0.843-1.012)	0.232	/	/		1.003(0.908-1.109)	0.947	/	/

SH, smoking history; AH, alcohol history; AKP, Alkaline phosphatase; LDH, Lactate dehydrogenase; AFU, α-L-fucosidase; SA, Serum sialic acid; FIB, Fibrinogen; TPSA, total prostatic specifific antigen; fPSA, free prostatic specifific antigen; f/T, free/total prostatic specifific antigen ratio; PCa, prostate cancer; CSPCa, clinically signifificant prostate cancer, which was defifined as Gleason grade ≥ 2; OR, odds ratio; CI, confifidence interval.

**Figure 1:**
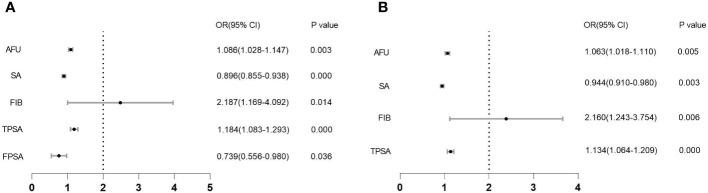
Forest map of multivariate regression analysis. **(A)**: Forest map between PCa and non-PCa; **(B)**: Forest map between CSPCa and non-CSPCa.

Similarly, in the CSPCa groups versus the non-CSPCa groups, factors other than TPSA were not significant in the univariate logistic regression analysis. The multifactor logistic regression, however, revealed statistical significance for AFU, SA, FIB, and TPSA (**Table 2**, all VIF < 5.000). We also plot forest maps of multivariate regression analysis as shown in **Figure 1**.

### 3.3 ROC curve analysis of variables

**Figure 2:**
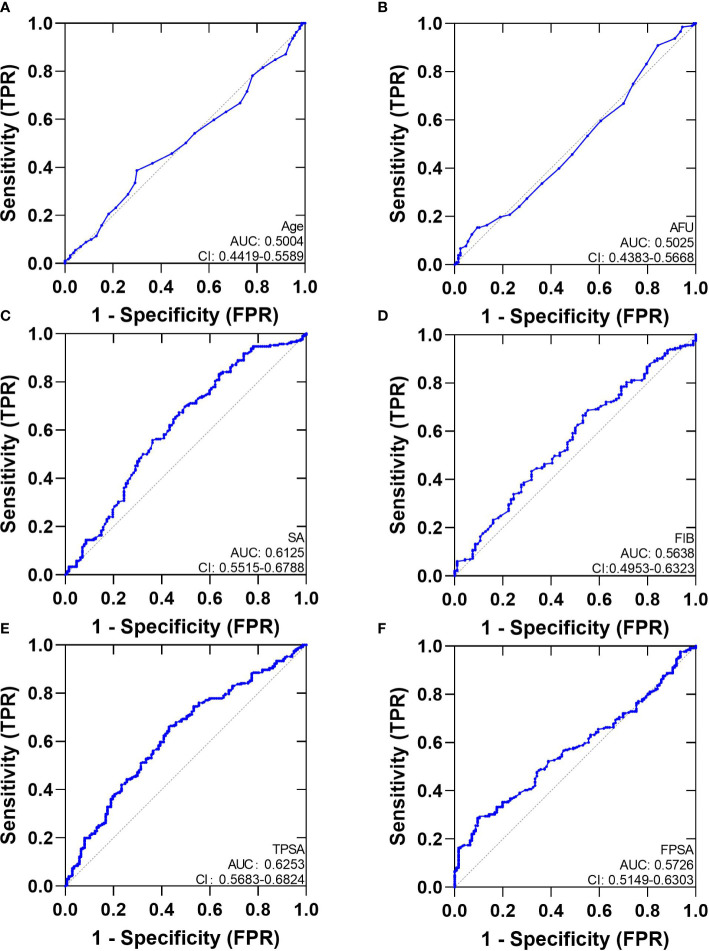
The AUC curves of Serum Biochemical Index, TPSA and FPSA. **(A)**: The AUC curves of Age; **(B)**: The AUC curves of AFU; **(C)**: The AUC curves of SA; **(D)**: The AUC curves of FIB; **(E)**: The AUC curves of TPSA; **(F)**: The AUC curves of FPSA.

**Figure 3:**
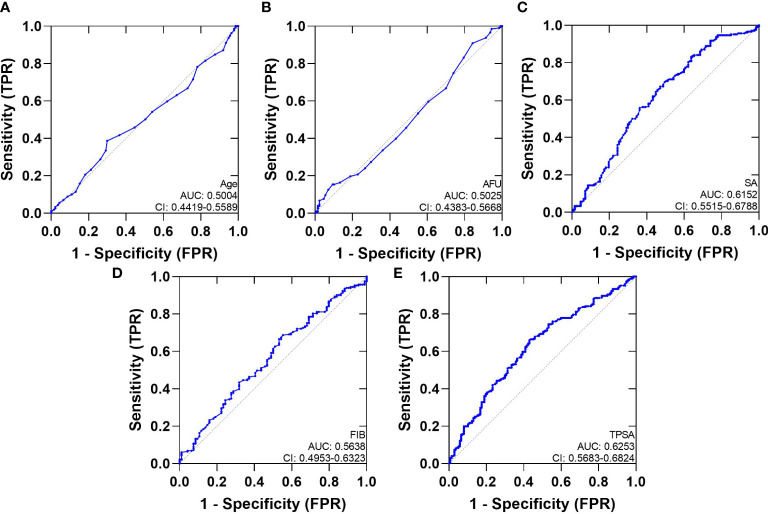
The AUC curves of Serum Biochemical Index and TPSA. **(A)**: The AUC curves of Age; **(B)**: The AUC curves of AFU; **(C)**: The AUC curves of SA; **(D)**: The AUC curves of FIB; **(E)**: The AUC curves of TPSA.

We analyzed ROC-AUC for Age, AFU, SA, FIB, TPSA, and FPSA to evaluate specific diagnostic variables for PCa and CSPCa. The detailed results of the analysis are presented in **Table 3**, **Figures 2**, **3**. As calculated using the parameters of the analysis, the TPSA had the highest predictive value for PCa values (AUC=0.6253, 95% CI: 0.5683–0.6824). The AUC values for the Age, AFU, SA, FIB, and FPSA were 0.5004 (95% CI: 0.4419–0.5589), 0.5025 (95% CI: 0.4383–0.5668), 0.6125 (95% CI: 0.5515–0.6788, 0.5638 (95% CI: 0.4953–0.6323), and 0.5726 (95% CI: 0.5149–0.6303), respectively. In the group of CSPCa, the ROC curve analysis showed that the AUCs of Age, AFU, SA, FIB, and TPSA were 0.5004 (95% CI: 0.4419–0.5589), 0.5025 (95% CI: 0.4383–0.5668), 0.6152 (95% CI: 0.5515–0.6788), 0.5638 (95% CI: 0.4953–0.6323) and 0.6253(95% CI: 0.5683–0.6824), respectively. As a result, TPSA can diagnose both PCa and CSPCa with the highest degree of certainty. Moreover, the SA level is one of the most powerful predictors, although not as effective as TPSA. In contrast, there is a lack of excellent predictive value for PCA and CSPCa using Age, AFU, FIB, and FPSA.

**Table 3:** ROC curve analysis of variables.

Variables	AUC	95% CI	Cut-Off	Sensitivity	Specifificity	Youden Index
PCa						
Age	0.5004	0.4419-0.5589	72.500	0.322	0.730	0.062
AFU	0.5025	0.4383-0.5668	11.500	0.909	0.157	0.066
SA	0.6152	0.5515-0.6788	42.850	0.966	0.047	0.013
FIB	0.5638	0.4953-0.6323	4.945	0.043	0.989	0.032
TPSA	0.6253	0.5683-0.6824	8.720	0.661	0.569	0.230
FPSA	0.5726	0.5149-0.6303	1.785	0.271	0.754	0.025
CSPCa						
Age	0.5004	0.4419-0.5589	72.500	0.323	0.696	0.019
AFU	0.5025	0.4383-0.5668	11.500	0.921	0.139	0.060
SA	0.6152	0.5515-0.6788	85.500	0.048	0.986	0.034
FIB	0.5638	0.4953-0.6323	2.995	0.574	0.518	0.092
TPSA	0.6253	0.5683-0.6824	9.015	0.697	0.526	0.223

AFU, α-L-fucosidase; SA, Serum sialic acid; FIB, Fibrinogen; TPSA, total prostatic specifific antigen; fPSA, free prostatic specifific antigen; PCa, prostate cancer; CSPCa, clinically signifificant prostate cancer, which was defifined as Gleason grade ≥ 2.

### 3.4 Development of a nomogram for PCa prediction

**Figure 4:**
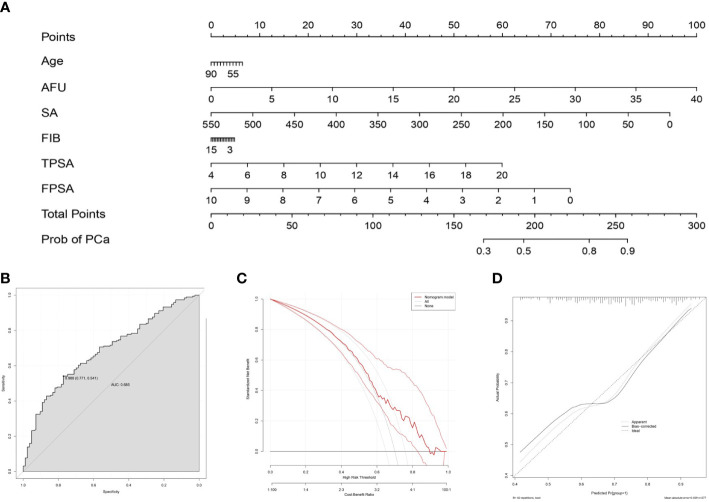
Nomogram for predicting PCa based on the training cohort. **(A)**: The prostate biopsy nomogram was developed in the training cohort, with age, AFU, SA, FIB, TPSA, and FPSA incorporated. ROC curve **(B)**, DCA curve **(C)** and calibration curve **(D)** for assessing the discrimination and calibration of the nomogram in predicting the probabilities of PCa.

To intuitively show the predictive value of the serum biochemical index for PCa, a nomogram was developed to predict the probability of PCa according to all significant factors for PCa and CSPCa occurrence (**Figure 4A**). In PCa prediction, the ROC curve showed good discrimination, and the AUC for the nomogram was 0.685 (CI:0.541-0.771) (**Figure 4B**). Moreover, the DCA curves and calibration curves revealed good agreement between predicted and observed PCa probabilities (**Figures 4C, D**).

## 4 Discussion

In this study, a retrospective analysis of prostate biopsies conducted on patients with PSA values ranging from 4.0 to 20.0ng/mL has been conducted. And we found that patients with PCa had significantly lower SA when compared with non-PCa patients. Further, the SA showed a high predictive value in the multivariable prediction model for PCa and CSPCa. AFU and FIB also demonstrated high predictive values in PCa and CSPCa multivariable prediction models, but they were not as accurate as SA in predicting PCa and CSPCa. In contrast, other serum biochemical indices, such as AKP and LDH, were insufficient for the diagnosis of PCa and CSPCa.

In more and more studies, it has come to light that serum biochemical-related cells and serum biochemical-related substances in cancer patients will undergo a series of changes as the disease progresses. These factors are closely related to the diagnosis and prognosis of cancers, such as AKP (18), LDH (19, 20), AFU (21), SA (21, 22), and FIB (23).

In the current state of prostate cancer screening, PSA is the most commonly used index (24, 25), Despite this, PCa and BPH were difficult to distinguish at PSA values between 4 and 20 ng/mL in some cases. As the gold standard, prostate biopsies required patients to tolerate greater pain, and it was possible that false negatives would result in microscopic prostate cancer not being detected. By contrast, the SA test had the advantage of being safe, low-cost, easy to implement, and generalizable in clinical settings.

As a terminal component of the non-reducing end of carbohydrate chains of glycoproteins and glycolipids, the yields of plasma SA typically increase during cancer progression. Sialic acid, while not a specific marker for one disease, has promise as a means to monitor disease progression and a measure of treatment effectiveness (26). The use of SA has been demonstrated to facilitate the growth of prostate cancer as well as bone metastases from the prostate in many studies (10, 12, 27, 28). SA has yet to be proven to be a predictive biomarker for PCa and CSPCa in patients with a PSA value between 4 and 20 ng/m, however. Ultimately, our findings suggest that PCa/CSPCa with a PSA value between 4 and 20 ng/mL detection is significantly correlated with a lower SA level. FIB and AFU are significant diagnoses for prostate cancer, but they cannot be used as effectively as SA for diagnosing PCa and CSPCa with PSA between 4.0 and 20.0 ng/mL.

**Figure 5:**
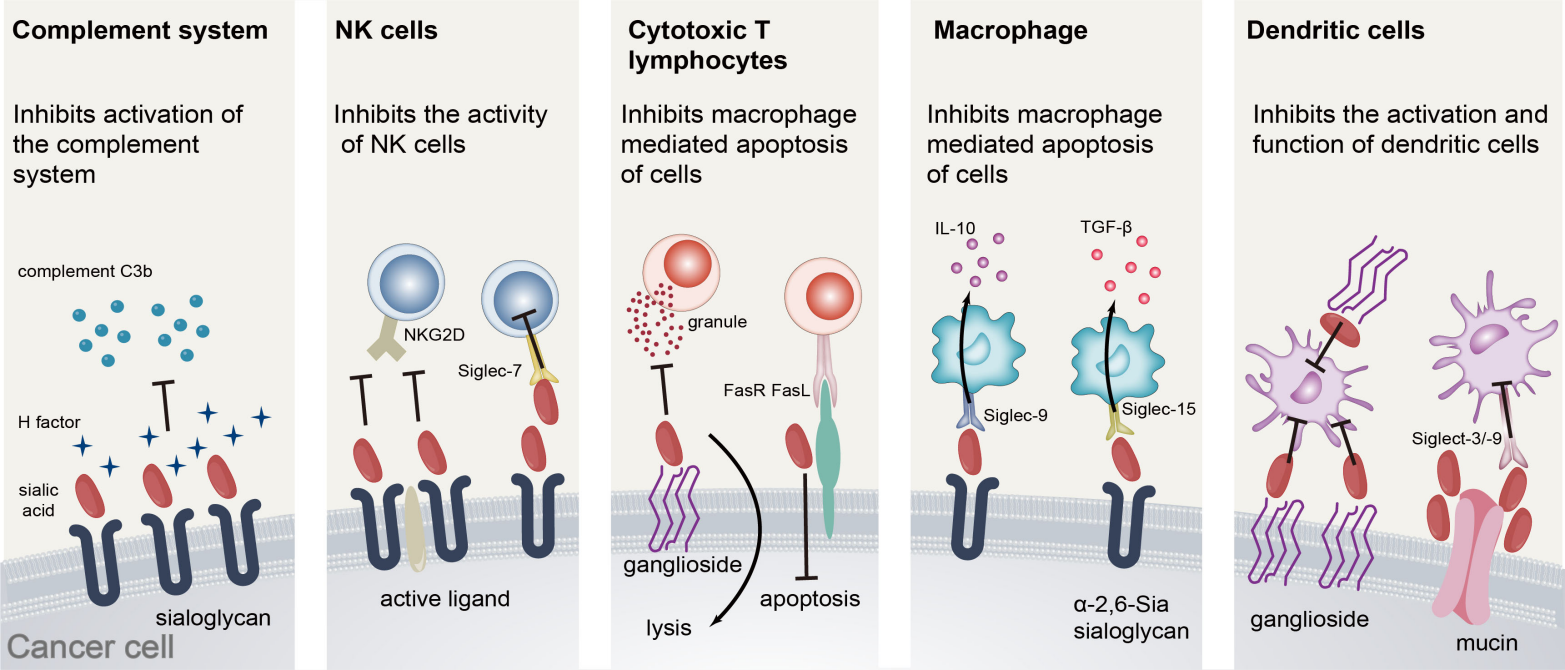
The mechanism of sialic acid promoting the occurrence and development of prostate cancer.

Our postulations regarding the role of SA in the metastasis of prostate adenocarcinoma primarily encompass the following factors (**Figure 5**). Firstly, the complement system constitutes a crucial component of innate immunity. Within this system, the H-factor serves as a pivotal regulatory protein (29). The anionic adhesion domain on factor H (30, 31) can selectively identify sialic acid or certain sulfated mucopolysaccharides and other detrimental molecules present on the surface of host cells. Upon binding to sialic acid on the surface of host cells, the H-factor effectively impedes the activation of the complement pathway, thus preventing any potential damage resulting from complement system activation (32). Furthermore, the sialylation of tumors serves to conceal active ligands on the surface of tumor cells that would otherwise bind to them. Additionally, sialylation on the surface of tumor cells can impede the formation of immune synapses between tumor cells and NK cells, thereby diminishing the cytotoxicity of NK cells towards tumors (33). This phenomenon could potentially be attributed to the incapacity of the activating receptor NKG2D on the surface of natural killer cells to identify activated ligands that have undergone modification by sialic acid on the surface of neoplastic cells (34–36), or to the substantial negative charge that sialic acid carries on the cell membrane surface. Additionally, the excessive expression of sialoglycans on the surface of malignant cells may facilitate evasion of immune surveillance by disrupting cytotoxic T lymphocyte activation and cytotoxicity mechanisms. Several pertinent studies have validated that gangliosides, specifically GD1a, present on the surface of tumor cells, can impede the transportation and exocytosis of cytoplasmic particles in CTL (37, 38), Consequently, this inhibition impedes the perforin, granzyme, and other contents of cytoplasmic particles from executing their function on target cells, thereby hindering the mediation of target cell death. Furthermore, the high sialylation of Fas on the surface of tumor cells can obstruct tumor cell apoptosis mediated by Fas and FasL (39, 40). Extant literature has established that the α-2,6 glycosidic bond-linked sialic acid present on the surface of lung cancer cells can stimulate the production of immunosuppressive cytokine TGF by Siglec-15 positive monocyte macrophages-β (transforming growth factor-β). Additionally, the sialoglycan on the surface of tumor cells impedes the secretion of TNF-α by Siglec-9 positive macrophages while promoting the secretion of IL-10. The co-expression of sialic acid on tumor cells and Siglec on macrophages plays a crucial role in determining the cytokine profile, thereby influencing the onset and progression of immune evasion in tumors. Furthermore, mucin, a high molecular weight glycoprotein, has been identified as a potential regulator of immune response. Specifically, studies have demonstrated that heavily sialylated mucins can bind to Siglecs on dendritic cells, leading to inhibition of their activation. Additionally, mucin 1 has been shown to promote the maturation of immature dendritic cells through aggregation. Nonetheless, these dendritic cells exhibit incomplete functionality, as they are unable to generate interleukin 12 (IL-12) or elicit an immune response from T cells (41, 42). Upon exposure to sialyltransferase, mucin 2 and sialic acid α-2,6 glycosidic bond linkage can attach to the surface of monocyte-derived dendritic cells via sigelec-3, leading to apoptosis induction. Furthermore, binding with sigelec-9 results in a reduction in IL-12 production. To conclude, sialic acid has the ability to facilitate tumor immune evasion through various mechanisms, including the attenuation of immune cell activity, disruption of complement system activation, and stimulation of immunosuppressive cytokine release.

The results of clinical examination can help clinicians to diagnose and treat diseases. But many external factors, such as the storage time of blood, the standard use of instruments, and so on, can affect the results and lead to errors. To avoid these contradictions and ensure the validity of the test results, we arrange the test sequence reasonably and test the blood sample within the best time. If the quality of the blood sample has changed as it has been stored longer, the blood will be collected again for retesting.

Nevertheless, a few limitations should be considered in this study. First, since our work was based on a retrospective study conducted at a single center, there may have been some statistically selective bias in the results. The second limitation is that despite our strict enrolling criteria, we were unable to completely exclude conditions like varicose veins in the lower limbs, atherosclerosis, and others which might impact SA levels. Additionally, because the sample size was limited, we did not distinguish between the biopsy strategies. Finally, all the data were collected from Qilu Hospital of Shandong University, so the results may have limitations in general application.

## 5 Conclusion

The SA is a significant predictor of PCa and CSPCa diagnoses in patients with PSA levels between 4.0 and 20.0ng/mL, according to our study. It is possible that in real clinical practice, they will help prevent unnecessary biopsies and biopsy-related morbidities. FIB and AFU had high predictive values for PCa and CSPCa, but they did not perform as well as SA in predicting these diseases.

## Data availability statement

The raw data supporting the conclusions of this article will be made available by the authors, without undue reservation.

## Ethics statement

The studies involving human participants were reviewed and approved by the Institutional Ethics Review Board of Qilu Hospital of Shandong University. Written informed consent for participation was not required for this study in accordance with the national legislation and the institutional requirements.

## Author contributions

JS performed the data analyses and wrote the manuscript. JS and LY participated in the collection of samples and clinical data. LY participated in the study design and revising of the manuscript. All authors have read and agreed to the published version of the manuscript.
